# Interventions to promote work ability by increasing physical activity among workers with physically strenuous jobs: A scoping review

**DOI:** 10.1177/1403494820917532

**Published:** 2020-06-09

**Authors:** Satu Mänttäri, Juha Oksa, Sirpa Lusa, Eveliina Korkiakangas, Anne Punakallio, Tuula Oksanen, Jaana Laitinen

**Affiliations:** Finnish Institute of Occupational Health, Finland

**Keywords:** Workplace interventions, physical activity, work ability, physical workload, scoping review

## Abstract

*Aims*: The potential benefits of workplace physical activity (PA) interventions are in improving both health and important workplace outcomes. Despite the differences in PA level between physically strenuous and inactive work, the literature reporting the effectiveness of the interventions does not usually differentiate physically active and inactive jobs. The aim of the current study was therefore to collect and synthesise research evidence on workplace PA interventions to promote work ability specifically among workers in physically strenuous jobs by means of a scoping review. *Methods*: The databases Medline, Cochrane Central and Scopus were used to identify interventions to promote work ability by increasing PA among workers in physically strenuous jobs. An iterative method was used to obtain an overview of the study elements and to extract details on the study design, sample, intervention, outcomes and effectiveness. *Results*: A total of 47 studies evaluating eight categories of interventions were found. Out of these, 18 reported significant effects on work ability. Positive results came from a range of different interventions, including aerobic exercise, strength training, combined aerobic exercise and strength training, stretching, yoga, consultation and tailored physical exercise programmes. ***Conclusions*: Few interventions were effective in promoting work ability by increasing PA among workers in physically strenuous jobs. In particular, trials based on the demands of work, multimodal interventions and applying wearable technology are needed**.

## Introduction

Workers in physically strenuous jobs, such as firemen and construction workers, are often exposed to intense, repeated or sustained exertion, unexpected peak loads and the need to maintain extreme and static body postures at work. In occupations with such physical demands, work ability is hence based to a considerable extent on workers’ physical capacity. When an individual’s physical capacity does not meet the demands of the job, the risk for poor work ability is increased [1].

Although workers in jobs involving physical stress are physically active for large parts of their work shifts, and the general health benefits of regular physical exercise are well known [2], physically demanding work does not seem to prevent a decline in work ability [3]. Similarly, occupational physical activity (PA) alone does not improve health (the PA paradox) [4,5]. A recent meta-analysis even suggests that moderate and high levels of occupational PA increase the risk of cardiovascular disease [6]. Nevertheless, the socio-economic as well as individual consequences of a high physical workload are substantial in terms of early retirement, sickness absence, musculoskeletal disorders and poor work ability [7–9].

Physical training is thought to improve work ability in physically demanding work by improving muscular strength and endurance [10]. It is recommended that adults undertake 30 minutes or more of moderate-intensity PA on most, preferably all, days of the week [11]. Since most adults spend many hours at work several days a week, the workplace provides a suitable setting to increase PA. There is growing evidence that workplace PA interventions can positively influence PA behaviour [12]. Yet, the variance of these studies evaluating workplace PA-promoting programmes is high, and well-designed studies assessing the impact of their implementations are still needed. In any case, supervised and group-based intervention protocols, often used at workplaces, seem to enhance exercise adherence compared to home-based exercise interventions [13].

There are several reviews and meta-analyses on workplace PA interventions aiming to classify and describe effective interventions that promote work ability [14–17]. However, despite the differences in PA level between physically strenuous and sedentary work, the literature reporting the effectiveness of the interventions does not necessarily differentiate physically active and inactive jobs. For this reason, and given the important preventive nature of PA but contradiction in terms of occupational PA health consequences, this scoping review was conducted to identify systematically the research done among workers performing physically demanding work tasks. The review followed the PRISMA-ScR format [18] to gather information on what is already known about this subject and to provide an indication on the knowledge gaps in the existing literature. It also aimed to assess the extent of the interventions studied and to recognise those that are effective.

The following research questions were formulated: What is known from the literature about the nature of workplace PA interventions provided for employees performing physically demanding jobs? Do the interventions consider the physical demands of the job? What are the gaps in research related to workplace PA aiming to enhance work ability?

## Methods

### Literature search

The databases Medline, Cochrane Central and Scopus were searched for studies related to interventions promoting PA at work or in leisure time to enhance work ability in physically strenuous jobs. The systematic search strategy consisted of keywords produced with an iterative process for work ability, pain, occupational health, training, physical work, exercise, PA, research and all relevant variations and synonyms.

### Criteria for considering studies for this review

Peer-reviewed journal papers were included to this review if they were published in 1980–2017, written in English and available in full-text electronic or hard-copy format. The titles and abstracts of each article were read by two reviewers to evaluate whether the manuscript met all of the following inclusion criteria: (a) outcome measure was work ability (self-report or objective), absence/absenteeism from work or presenteeism; (b) study was conducted in a workplace setting or organised by the employer, and participants were employed during the study; and (c) any intervention study (pre–post, controlled and/or randomised) that aimed to increase the amount of PA at work or in leisure time. The articles were then divided into two categories according to work intensity following Ainsworth’s Compendium of Physical Activities [19]: (a) mainly sedentary work without any other kind of movement besides sitting, corresponding to the energy metabolism of one metabolic equivalent (MET) and (b) work with diverse movements (not solely sitting) likely to increase energy metabolism above one MET and, in this context, defined as strenuous work. This review focuses on the latter. Conflicts during the inclusion process were discussed to reach a consensus.

### Data extraction and management

Data were extracted into a data-charting form in which the following details were listed: study ID, country, year of study, branch of industry, objective of the study, study design, number of participants and follow-up time. For the study participants, small business entrepreneurs, selection process, sex, age, state of health, occupation, type of work and work intensity were extracted. For type of intervention(s), taking place at work/in leisure time, description of the intervention (type), compliance, theory, elements, frequency and duration, resource use, costs, comparison intervention and provider were extracted. For the outcomes, primary and secondary outcomes, measurement instrument, validation, beneficial and harmful effects were extracted. In addition, related research articles were listed.

To refine the inclusion of studies and the findings, new broader categories were established summarising the available evidence and providing a synthesis of the results. The approach is comparable to finding common themes in summarising qualitative research [20].

### Data analysis

Based on the findings from the studies, recommendations for future systematic reviews were formulated. Gaps in primary research concerning PA-increasing interventions and developed recommendations for future primary studies were listed.

## Results

**Figure 1.**
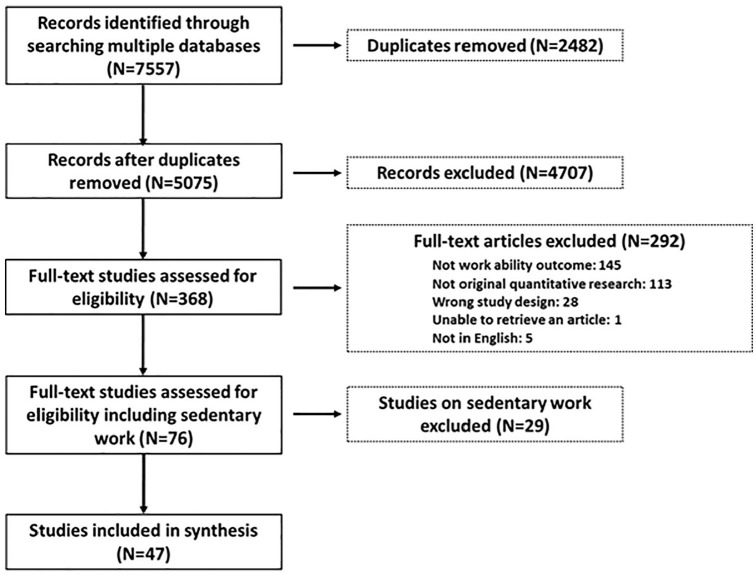
PRISMA flow chart showing process of selecting papers for scoping review.

The process followed in the selection of papers for this review is shown in Figure 1. After duplicates were removed, 5075 citations were identified from searches of the electronic databases. Based on the title and the abstract, 4707 were excluded, with 368 full-text articles being retrieved and assessed for eligibility. Of these, 292 were excluded for the following reasons: 145 had outcomes that were not the focus of this review (e.g. occurrence of musculoskeletal disorder or cardiovascular disease alone as an outcome), 113 were not considered to be original quantitative research (e.g. review articles, pilot studies, protocols, extended abstracts, etc.) and 28 had a study design or setting that did not meet the eligibility criteria (e.g. students as study subjects, non-controlled studies). Five studies were excluded due to language and one because it was not achievable. Out of the remaining 76 studies, 47 were done among workers with physically strenuous jobs and were therefore considered eligible for this review. The characteristics of these studies are presented in Table I.

**Table I.** Characteristics of included studies (*N*=47) evaluating interventions to promote work ability by increasing physical activity among workers with physically strenuous jobs.

Study ID	Country	Year	Study design	***N***	Participants	Intervention	Control	Primary outcome	Duration of the study
Andersen	Denmark	2012	RCT	537	Industrial technicians	Resistance training	Usual practice	MSP	20 weeks
Barene	Norway	2014	RCT	118	Hospital employees	Soccer and Zumba	Usual practice	Physical fitness	12 weeks
Beach	Canada	2014	RCT	60	Firefighters	FIT+MOV groups	Usual practice	Physical fitness	12 weeks
Brandt	USA	2015	RCT	13	Helicopter crew members	Self-administered exercise	Usual practice	MSP	12 weeks
Brox	Norway	2005	RCT	119	Nursing home employees	Light exercise	Usual practice	Absence, HRQoL	6 months
Chaleat-Valayer	France	2016	RCT	342	Health-care workers	Training sessions	Usual practice	LBP	2 years
Christensen	Denmark	2013	RCT	144	Health-care workers	Strength exercise	Usual practice	Presenteeism, Absenteeism	1 year
DeBoer	Netherlands	2004	CBA	116	Electronic equipment manufacturers	Psycho-medical assessment and consultation	Usual practice	Early retirement	2 years
Dehlin	Sweden	1981	CBA	45	Nursing aides	Physical training	Usual practice	LBP	8 weeks
Dongen	Netherlands	2013	RCT	730	Hospital workers	Yoga	Usual practice	Cost effectiveness	1 year
Edries	South Africa	2013	RCT	90	Textile workers	Aerobic, core strength training, stretching	Usual practice	HRQoL	6 weeks
Eriksen	Norway	2002	RCT	860	Postal workers	Physical exercise	Usual practice	Absence	1 year
Gamble	Ireland	1993	CBA	14	First responders	Indoor soccer, circuit training, flexibility exercises	Usual practice	Physical fitness	10 weeks
Gerdle	Sweden	1995	CBA	97	Home care workers	Coordination, strength, aerobic and stretching exercises	Usual practice	MSP, physical fitness	1 year
Gram	Denmark	2012	RCT	67	Construction workers	Aerobic and strength training	Usual practice	MSP	12 weeks
Gram	Denmark	2016	RCT	67	Construction workers	Aerobic and strength training	Usual practice	Physical fitness	12 weeks
Gundewall	Sweden	1993	RPS	69	Nurses and nurse’s aides	Back muscle dynamic endurance and isometric strength exercises	Usual practice	MSP, physical fitness	13 months
Hamberg-vanReenen	Netherlands	2009	RCT	22	University workers	Resistance training	Usual practice	MSP, physical fitness	8 weeks
Han	South Korea	2016	CBA	100	Assembly line workers	Hamstring stretching exercises with and without pelvic control	Usual practice	LBP	6 weeks
Horneij	Sweden	2001	RPS	282	Nursing aides or assistant nurses	Physical training programme	Usual practice	LBP, physical load	18 months
Jørgensen	Denmark	2011	RCT	294	Cleaners	Physical coordination and cognitive–behavioural training	Usual practice	WAI, absence	1 year
Jakobsen	Denmark	2015a	RCT	200	Healthcare workers	Strength training	Other intervention	Physical load, NfR	10 weeks
Jakobsen	Denmark	2015b	RCT	200	Healthcare workers	Strength training	Other intervention	WAI	10 weeks
Klatt	USA	2015	CBA	34	ICU personnel	Mindfulness in motion, yoga	Waiting list	Resiliency	8 weeks
Korshøj	Denmark	2015	RCT	116	Cleaners	Aerobic exercise	Lectures	Physical fitness	40 months
Kruger	Germany	2015	RCT	14	Welders	Strength training	Usual practice	Physical fitness	12 weeks
Nurminen	Finland	2002	RCT	260	Laundresses	Moderate exercise	Usual practice	WAI	15 months
Nygaard Andersen	Denmark	2015	RCT	54	Health care workers	Aerobic fitness and strength training	Usual practice	Absence	3 months
Park	USA	2017	RCT	66	Surgeons	Stretching micro breaks	Usual practice	MSP	2 weeks
Perkiö-Mäkelä	Finland	1999	RCT	126	Farmers	Physical exercise	Usual practice	Physical fitness, MSP, WAI	3 years
Perkiö-Mäkelä	Finland	2001	RCT	126	Farmers	Physical exercise	Usual practice	Physical fitness	6 years
Perkiö-Mäkelä	Finland	1999	RCT	126	Farmers	Physical exercise	Usual practice	Physical fitness	1 year
Pohjonen	Finland	2001	NCT	87	Home care aids	Physical exercise	Usual practice	Physical fitness	5 years
Rantonen	Finland	2012	RCT	176	Employees in a forestry company	Progressive back-specific exercises	Usual practice	MSP, HRQoL, absence	4 years
Roussel	Belgium	2015	RCT	69	Nurses, caregivers, physio- and occupational therapists	Exercise	Usual practice	LBP, absenteeism	6 months
Strijk	Netherlands	2012	RCT	730	nr	Exercise	Usual practice	Physical fitness	6 months
Strijk	Netherlands	2013	RCT	730	nr	Exercise	Usual practice	Vitality	12 months
Sundstrup	Denmark	2014	RCT	66	Slaughters	Strength training	Other intervention	MSP	10 weeks
Sundstrup	Denmark	2014	RCT	66	Slaughters	Strength training	Other intervention	Primary: work ability	10 weeks
Tucker	USA	2016	RCT	40	RNs, MAs	Workstation treadmill, Wii	Other intervention	Physical fitness, absence	6 months
Tveito	Norway	2009	RCT	40	Nurses, nursing auxiliaries, assistants	Physical exercise	Usual practice	Absence	9 months
Viester	Netherlands	2015	RCT	314	Construction workers	Lifestyle coaching	Usual practice		1 year
Vingard	Sweden	2009	RCT	370	Nurses, care workers, kitchen workers	Physical fitness Programme including different exercise options	Usual practice	MSP	3 years
Von Thiele Schwarz	Sweden	2011	RCT	177	nr	Exercise of medium-to-high intensity	Usual practice	WAI	12 months
Von Thiele Schwarz	Sweden	2008	RCT	177	nr	Exercise of medium-to-high intensity	Usual practice	Physical fitness	12 months
Warming	Denmark	2008	RCT	181	Nurses	Physical fitness training	Usual practice	LBP, absence	12 months
Zavanela	Brazil	2012	RCT	96	Bus drivers	Resistance training	Usual practice	BP, absenteeism	36 weeks

RCT: randomised controlled trial; CBA: controlled before-and-after study; RPS: randomised prospective study; NCT: non-randomised controlled trial; nr: not reported; HRQoL: health-related quality of life; LBP: low back pain; MSP: musculoskeletal pain; WAI: work ability index; NfR: need for recovery.

Of the 47 included studies, 46 reported data from multiple groups, and one reported data from a single study arm only (crossover study design). Thirty-eight (81%) studies were some form of randomised controlled trial (RCTs), six were controlled before-and-after studies (CBAs), two were randomised prospective studies (RPSs) and one was a non-randomised controlled trial (NCT).

Twelve studies were conducted in Denmark, seven in Sweden, six in both Finland and the Netherlands, four each in Norway and the USA and one each in Belgium, Brazil, Canada, France, Germany, Ireland, South Africa and South Korea. Thus, 62% of the studies were conducted in the Nordic countries.

Sample sizes varied from 13 to 860 subjects representing employees in the following branches of industry: health care (e.g. nurses, paramedics, dentistry), safety (e.g. helicopter crew members, firefighters), industry (e.g. food industry, metallurgy, construction), transportation (e.g. bus drivers) and others (e.g. postal service, manual handling). Of these, two studies were done among SB entrepreneurs (<50 employees). One study had a criterion that only companies employing more than 50 workers were included [21]. The type of work was assessed as light (<3 METs) in five studies, moderate (3–6 METs) in four studies and heavy (>6 METs) in 14 studies. In two studies [22,23], the work intensity was measured, and it was high in both cases (metabolic workload >33% VO_2_max). In 22 studies, the work intensity was not reported.

The interventions used to promote work ability by increasing PA among workers with physically strenuous jobs were categorised as (a) aerobic exercise (e.g. aerobic, Zumba; *n*=4), (b) strength training (e.g. resistance training, job-specific training, stabilising exercises; *n*=9), (c) combined aerobic exercise and strength training (*n*=22), (d) stretching (*n*=2), (e) non-traditional PA (e.g. mindfulness in motion, yoga; *n*=4), (f) eTraining and mobile coaching (e.g. Wii, SMS, DVD; *n*=2), (g) tailored physical exercise programmes (*n*=3) and (h) consultation (*n*=1). Just over two-thirds (68%) of the interventions were performed at the workplace. In three of the interventions [21,24,25], job-specific physical demands were taken into account.

The instruments used to measure outcomes were questionnaires, statistics (registry) and objective physiological measurements (Table II). Those used most often were Work Ability Index (WAI) questionnaires measuring work ability (*n*=14) and various physiological methods measuring physical performance (*n*=14). The Nordic Musculoskeletal Questionnaire was used as a scale for both absenteeism (in one study) and pain-related work ability (in three studies). Pain was also measured with a visual analogue scale in three studies, with the Numerical Pain Rating Scale in one study and with a modified questionnaire in six studies. PA was measured with an accelerometer in one study, with a diary/questionnaire in five studies and with a wearable monitor in one study. Cost effectiveness in one study was measured using economic calculations.

**Table II.** Characteristics of outcome measurements of interventions to enhance work ability (*N*=47).

Outcome type	Outcome measurement	***n***
Work ability	WAI questionnaire	15
Absence	Sickness absence days, records	8
Absenteeism	NMQ (related to LBP)	1
Presenteeism	STEM questionnaire	1
Pain	VAS scale, NMQ, NPRS, modified questionnaire	14
Aerobic capacity	Ergometer, VO_2_max, treadmill, UKK walking test, diverse physical testing, HR	15
Musculoskeletal	Grip strength, Shirado test, Sorensen test, maximal force, EMG, FCT, isometric strength	12
Physical activity	Accelerometer, questionnaire/diary, sense-wear monitor	7
Cost effectiveness	Economic calculations	1

WAI: work ability index; NMQ: The Nordic Musculoskeletal Questionnaire; LBP: low back pain; STEM: a questionnaire used in STEM (statens energimyndighet, Swedish) study; VAS: visual analogue scale; NPRS: numerical pain rating scale; UKK: a test developed by the Urho Kaleva Kekkonen Institute for Health Promotion Research (the UKK Institute); HR: heart rate; EMG: electromyography; FCT: functional capacity test.

Five of the included studies were described as theory based. In the study by Edries et al. [26], the aim was to evaluate the short-term effect of a wellness programme on health-related quality of life, health behaviour change, body mass index and absenteeism. The wellness programme intervention used was based on the principles of cognitive–behaviour therapy (CBT). CBT-based programmes aim to equip individuals with the knowledge, behavioural ability and cognitive skills needed to improve their state of health and functional abilities [27]. Roussel et al. [28] implemented an intervention based on the theoretical prevention model [29] in order to evaluate the effectiveness of a multidisciplinary prevention programme for low back pain. In three of the included studies [30–32], intervention mapping (IM) was used. IM is a health-promotion protocol for selecting and applying social and behavioural science theories, such as theories of health psychology, to the planning, implementation and evaluation of health-promotion programmes. In the studies by Strijk et al. [31,32], the objective of evaluating the effectiveness of workplace vitality intervention on PA, fruit intake, aerobic capacity, mental health and need for recovery was based on IM. In Viester et al. [30], IM was used in a lifestyle coaching intervention aiming to reduce musculoskeletal symptoms and sickness absence, and to improve work ability, work-related vitality and work performance. None of the studies that used a theory-based approach reported a significant effect of the intervention on work ability or absenteeism.

Only 18 (38%) studies found a significant increase in work ability (Table III). In these studies, strength training (*n*=5) or combined aerobic and strength training (*n*=6) were the most commonly used interventions. One study [21] with a significant effect on work ability (reduced aerobic workload) used an aerobic exercise intervention tailored for each workplace individually. However, despite the positive improvement in work ability, this study was the only one reporting an apparent adverse event, that is, potential cardiovascular overload due to increased systolic blood pressure.

**Table III.** Characteristics of interventions having a significant effect on work ability.

Study ID	Sample	Study design	Intervention	Significant beneficial/harmful effects	Duration/follow-up
			*Aerobic exercise*		
Korshøj et al. 2015 [21]	116 cleaners, mean age 45.3 years, 75.9% female	RCT	Supervised aerobic exercise, intensity >60% VO_2_max, tailored for each attending company, reference (*N*=59) and intervention (*N*=57) groups	Improvement in cardiorespiratory fitness, decrease in aerobic workload, and resting and sleeping HR/increased systolic BP	4 months
			*Strength training*		
Andersen et al. 2012 [7]	537 industrial technicians, mean age 43.5 years, 25% female	RCT	Supervised specific resistance training for the shoulder, neck and arm muscles, reference (*N*=255) and intervention (*N*=282) groups	Reduced pain intensity and work disability	20 weeks
Jakobsen et al. 2015 [52]	200 health-care workers, mean age 42.0 years, all female	RCT	Either workplace or home-based supervised and self-regulated strength training, physical exercise at work (*N*=111), physical exercise at home (*N*=89)	Reduced physical exertion and need for recovery when physical exercise was performed at workplace (more effective compared to home-based exercise)	10 weeks
Jakobsen et al. 2015 [53]	200 health-care workers, mean age 42.0 years, all female	RCT	Either workplace or home-based supervised and self-regulated strength training, physical exercise at work (*N*=111), physical exercise at home (*N*=89)	More effective prevention of deterioration in work ability when performing physical exercise at the workplace compared to home-based exercise	10 weeks
Kruger et al. 2015 [25]	14 welders, mean age 28.3 years, all male	RCT	Supervised strength training programme specifically designed to address those muscles mainly used during various welding positions, reference (N=7) and intervention (N=7) groups	Reduction of the relative muscle load employed during welding	12 weeks
Zavanela et al. 2012 [34]	96 bus drivers, all male	RCT	A periodised resistance training programme implemented within the workplace, reference (*N*=48) and intervention (*N*=48) groups	Reduction in BP and pain incidence, improvement in muscle endurance and flexibility, lowered work absenteeism rate	24 weeks
			*Combined aerobic and strength training*		
Gamble et al. 1993 [23]	14 ambulance-men, all male	CBA	Indoor soccer and circuit training (aerobic and strength), reference (*N*=6) and intervention (*N*=8) groups	Increase in flexibility, leg and abdominal muscle power and VO_2_max. Decrease in %VO_2_max and HR in simulation, i.e. increase in work capacity.	10 weeks
Gundewall et al. 1993 [54]	60, mean age 37.5 years, 98% female	RCT	Back muscle dynamic endurance and isometric strength exercises, reference (*N*=28) and intervention (*N*=32) groups	Decrease in lost working days, complaints due to LBP and in pain intensity; increase in back muscle strength	13 months
Pohjonen et al. 2001 [33]	87, mean age 42.6 years, all female	CT	Physical exercise (aerobic and strength training), reference (*N*=50) and intervention (*N*=37) groups	Improvement in physical fitness and perceived health status; prevention of early decline in work ability	9 months/1 and 5 years
Vingård et al. 2009 [55]	370, all female	RCT	Physical fitness programme including different exercise options, reference (*N*=165) and intervention (*N*=205) groups	Experienced improvements in general health and work ability, unchanged physical workload	36 months
von Thiele Schwarz et al. 2011 [50]	177 dental health-care workers, mean age 46.6 years, 85.9% female	RCT	Intervention group 1: exercise of medium-to-high intensity (*N*=61), intervention group 2: reduced workhours (*N*=51), reference group (*N*=65)	Improved productivity (the production levels, i.e. number of treated patients, and the self-rated productivity increased despite fewer work hours)	12 months
			*Other physical activity*		
Han et al. 2016 [56]	100 automotive parts assembly line workers, mean age 38.5, both sexes	CBA	Hamstring stretching exercises with (*N*=34) and without (*N*=34) pelvic control, reference group (*N*=32)	Reduction in pain intensity, increase in work ability index	6 weeks
Klatt et al. 2015 [24]	34 workers in Intensive Care Units (ICUs),	CBA	Mindfulness in motion, yoga, wait-list control and intervention groups	Increase in resiliency	8 weeks
Park et al. 2017 [57]	66 surgeons and operating room staff, mean age 47.0, 31% female	RCT	Stretching micro breaks, standardised exercises	Improved surgeon post-procedure pain scores in all anatomic regions in both open and minimally invasive cases, improvements in physical performance	2 days
			*Consultation or tailored physical programmes*		
De Boer et al. 2004 [58]	116 electronic equipment manufacturers, mean age 53.4, 7% female	RCT	Psycho-medical assessment and consultation, three sessions reference (*N*=55) and intervention (*N*=61) groups	Improved work ability and quality of life index; less burn-out	6 months/2 years
Horneij et al. 2001 [59]	282 nursing aides or assistant nurses, mean age 44.0, all female	RCT	Intervention group 1: individually tailored physical training programme (IT, *N*=90); intervention group 2: stress management (SM, *N*=93), reference group (*N*=99)	Reduced perceived physical exertion at work for IT, reduction in LBP for IT and SM	18 months
Sundstrup et al. 2014 [60]	66 slaughters, mean age 45.5, 23% female	RCT	Intervention group 1: strength training locally for the shoulder, arm and hand muscles (*N*=33); intervention group 2: ergonomic training (*N*=33)	Clinically relevant improvements in pain, work disability and muscle strength in response to customised resistance training at the workplace	10 weeks
Sundstrup et al. 2014 [61]	66 slaughters, mean age 45.5, 23% female	RCT	Intervention group 1: strength training locally for the shoulder, arm and hand muscles (*N*=33); intervention group 2: ergonomic training (*N*=33)	More effective prevention of deterioration of work ability among workers with chronic pain and work disability with specific strength training at the workplace	10 weeks

A follow-up study by Pohjonen et al. [33] showed that in addition to improving physical fitness, perceived health status and work ability immediately after the intervention, workplace physical exercise intervention also prevents an early decline in work ability. Among those studies reporting significant results, only one study [34], besides the previously mentioned study by Pohjonen et al. [33], had a follow-up period after the intervention period. Overall, 8/47 studies included had a follow-up period of more than six months but less than or equal to 12 months, whereas six studies had a follow-up period of more than 12 months, with six years being the longest. In general, few studies followed the effect of the intervention after it ceased, and in most cases the effect did not last.

## Discussion

In this scoping review, 47 primary studies evaluating workplace interventions to enhance work ability in physically strenuous jobs were identified. Eight types of interventions were identified that increased PA among workers with physically strenuous jobs: aerobic exercise, strength training, combined aerobic exercise and strength training, stretching, non-traditional PA (e.g. yoga), eTraining and mobile coaching, tailored physical exercise programmes, and consultation. The WAI and objective measures to assess physical loading were most often used instruments to measure the outcomes. The study authors reported a significant and relevant effect of the intervention in 18/47 studies. Out of five studies that used a theory-based approach, none reported a significant effect of the intervention on the outcome measure (i.e. work ability or absenteeism). One of the studies reported a harmful effect of the intervention [21]. Few studies used a follow-up period after the intervention ceased [33,34].

Some gaps were identified in the evidence. First, only one paper reported adverse events after the implementation. Generally, the literature on the undesirable side effects of health-promotion studies is inadequate, although it is common knowledge that interventions, in other fields besides the medical field, can have adverse effects [35]. Since workplace exercise generally improves fitness and thus has beneficial effects [17], potential harmful effects should also be measured to avoid misinterpretation of the absolute advantages of the intervention. In recommendations, especially for workers with high occupational PA, the potential cardiovascular overload from additional aerobic exercise should be taken into consideration.

Second, few papers were follow-up studies, even though it is only with more long-term follow-up in intervention studies (e.g. beyond 12 months) that it will be possible to assess the accuracy of the estimates on the maintenance of changes following the intervention. Health-related interventions should thus be continually and intensively provided and followed to attain long-term effects.

Third, based on the evidence, intervention studies [21,24,25] that have taken into account the physical demands of work are limited. The demands should be met by workers’ physical capacity, and these two factors should be considered as key points when designing interventions. In fact, job-specific training proved to be effective in the included studies, since all three studies using tailored interventions reported significant effects on work ability. On the other hand, one of the tailored interventions [21] was the only included study reporting adverse side effects. In Korshøj et al. [21], aerobic exercises were tailored to each of the enrolled cleaning companies individually in order to meet the requirements of feasibility and motivation. The modified intervention mapping approach (i.e. intervention tailored specifically to the individual needs and wishes of the participating company) was considered a strength of the study leading to significantly decreased aerobic workload. To meet the requirements of another group of manual workers (welders), a strength training programme was specifically designed to address those muscles mainly used during various welding positions [25]. The training-induced increase in muscular strength was translated into improved working ergonomics, significantly improving work ability. In Klatt et al. [24], an intervention of gentle yoga stretches, in addition to mindfulness meditation, was targeted at employees working in high stress environments. Work engagement and resiliency were evaluated, and both increased significantly. There has been a trend towards a reduction in total and occupational PA over the past decades [36]. Despite this, physical demands in work life remain high, and the proportion of the global workforce with a high physical workload is substantial [37]. Since exposure to physically strenuous job tasks evidently increases the risk for long-term sickness absence, early retirement and the risk of receiving disability pension [38], attention should be paid either to reducing the physical workload or improving work ability. Therefore, it is suggested that the physical demands of work should consistently be taken into consideration and, more importantly, quantitatively measured when a training programme intervention is carried out at the workplace, as was done in the previously described studies.

Fourth, only two papers reported technology-enhanced solutions, that is, DVD-based strengthening exercises [39] and mobile health coaching via text messaging [40]. Wearable technology, such as activity monitors or fitness trackers, was not used in enhancing work ability by increasing PA although interventions using computer, mobile and wearable technologies appear to be effective in improving health and reducing sedentary behaviour [41]. This may be due to the lack of thorough validation studies for these devices. Since wearable technology has become very popular, increasingly cheaper and provides convenient monitoring of various parameters, implementations applying modern, validated wearable technology are needed to address this gap in knowledge.

Fifth, besides interventions designed according to work demands, interventions directed at workers in small organisations are sparse. Small and microenterprises (<10 employees) account for >95% of firms and 60–70% of employment in, for example, the European Union and therefore represent a substantial part of the workforce being in a unique position when it comes to occupational safety and health. Consequently, targeted interventions for workers in small businesses are also needed, especially due to the lack of support from occupational health services, the occupational health and safety administration and human resources [42].

Sixth, since the interventions used aimed to increase PA, the natural result was often improvement in physical fitness and therefore a decrement in workload. Good physical fitness levels also advance recovery and hence may have a positive secondary effect on work ability [43]. In the present review, the level of recovery was assessed in 9% of the papers. The role of recovery from heavy muscular work is important, since fatigue, especially when accumulating, may cause musculoskeletal symptoms and disorders and, further, early retirement [44]. Therefore, it is recommended that elements aiming to improve recovery from physical work are also included in workplace intervention studies, and that the outcomes related to recovery are also measured.

A comprehensive approach was taken to scoping a variety of references to synthesise what is known about workplace interventions to enhance work ability in physically demanding jobs. The exhaustive inclusive process such as searching for sources from several databases and having two reviewers for every full text has added rigor to the scoping process and thus serves as strength of this paper. Furthermore, by extracting data comprehensively and by using a qualitative approach to synthesise the issues, comparison of the included studies according to many aspects was possible. However, some limitations do exist. The definition of work ability is not unambiguous, especially when physically strenuous work is being reviewed. Based on the extensive data of the Health 2000 study, work ability is defined by health, work, knowledge and skills [45]. The relationship between knowledge and skills and work ability was emphasised for well-educated individuals, people with physically undemanding jobs and people enjoying good health. The study also highlighted the fact that due to the diversity of work ability, measuring it is challenging. In the literature, there are two complementary deﬁnitions for work ability [46]. In the first sense, having work ability means having the occupational competence, the health required for the competence and the occupational features that are required for managing the work tasks. In the second sense, having work ability is having the health and the basic standard competence for managing some kind of job. Therefore, a comprehensive search protocol, analysis and unequivocal interpretation for work ability were difficult to conduct. Some outcomes of the intervention studies thus might not precisely refer to the work ability defined in the literature, or might affect work ability indirectly as in the case of decreased physical workload due to improved fitness levels. In addition, employment status was not controlled for, and therefore working hours per week, for example, may vary between the included studies.

Due to the comprehensive and far-reaching effects of work ability on well-being, health status and quality of life, there is a clear need for high-quality research of the interventions aimed to enhance work ability in physically intensive jobs. In addition, systematic reviews on the effectiveness of the interventions are also called for. In particular, it might be useful to examine further how the interventions aimed to increase PA at the workplace contribute to the PA health paradox [4,5]. For example, since workers with low cardiorespiratory fitness have an increased risk for cardiovascular disease from high occupational activity [6], a specific training programme reducing aerobic workload could be effective. In addition, since one of the proposed reasons for the PA health paradox is the nature of occupational PA [4], interventions breaking the occupational activity patterns during the work shift should be evaluated. Even though workplace fitness and health intervention programmes have become common [47], there are many limitations and problems within the methodology such as bias, lack of validation and poor statistical analyses. Studies of workplace PA interventions are often biased due to self-selection (e.g. Andersen et al. [7]). Also, awareness of the intervention can lead to dilution of the contrast between intervention and control groups (e.g. Nurminen et al. [48]). Thus, the ideal design of an intervention programme is RCT because it protects best against confounding and selection bias.

In the included studies, the interventions were often targeted at individuals even though the activity was performed in groups. The population effect is, however, often limited due to low adherence or a high drop-out rate. In the present data, the drop-out rate was on average 23%, varying from 0% to 48%, highlighting the difficulties in implementing effective interventions at the workplace. According to Wanzel et al. [49], the participation rate of fitness programmes rarely exceeds 20–40% of the employees. Therefore, new intervention approaches are needed to motivate workers to continue the intervention and especially to maintain their PA over time. One of the motivators that came up in the present data was a reduction in weekly working hours with mandatory physical exercise (e.g. Von Thiele Schwarz and Hasson [50] and Vingård et al. [51]). The current review thus suggests that physical activities during paid working hours are better investments to attain and maintain health and work ability compared to activities outside working hours.

From the sources reviewed for this scoping review, it can be understood that multifaceted interventions, such as intervention programmes consisting of ergonomic training, equipment modification and daily exercise, should be acknowledged to establish a significant change in work ability (e.g. Jakobsen et al. [52,53]). In addition, the present report indicates that in order to significantly improve work ergonomics and tolerance against the exposure to strenuous tasks, specifically designed tailored interventions meeting the requirements of the work tasks should be favoured (e.g. Viester et al. [30] and Wanzel [49]).

## Conclusions

This scoping review described the existing literature about research on workplace PA interventions provided for employees performing physically demanding jobs to promote work ability. Just 47 relevant papers were found, and out of these, 18 reported a significant and relevant effect of the intervention used. The majority of the interventions thus failed to show enhancement in work ability, although physical training naturally improved physical fitness. There is, however, insufficient evidence to evaluate the effectiveness of the interventions, and therefore more high-quality research that addresses the current lack of understanding, as well as systematic reviews, are needed. In addition, more focus should be given to microenterprises, interventions considering the physical demands of work, long-term follow-up and applying wearable technology.
